# Low gestational age at birth and difficulties in school—A matter of ‘dose’

**DOI:** 10.1371/journal.pone.0198482

**Published:** 2018-06-20

**Authors:** Rikke Wiingreen, Gorm Greisen, Jannet Svensson, Bo Mølholm Hansen

**Affiliations:** 1 Department of Neonatology, Copenhagen University Hospital, Rigshospitalet, Copenhagen, Denmark; 2 Department of Paediatrics, Herlev Hospital, Herlev, Denmark; King’s College London, UNITED KINGDOM

## Abstract

**Objectives:**

Several studies suggest a relationship between gestational age at birth and risk of school difficulties. Our study aimed to investigate the association between the entire range of gestational ages and significant school difficulties measured as 1) More than nine hours per week special educational support and 2) Failing to complete compulsory school.

**Methods:**

A population-based register study including all children attending the Danish compulsory school in 2015/2016 and all live-born infants born in Denmark from 1992 to 1997. Data were collected and linked using multiple registers held by Statistic Denmark. Multiple logistic regression analyses were used to estimate the association between gestational age and significant school difficulties, adjusted for explanatory variables.

**Results:**

For measurement 1) “Special educational support” 615,789 children entered the analyses after exclusion of those with missing neonatal data. The risk of special educational support increased gradually across the entire range of gestation from 40 to ≤24 weeks: The adjusted odds ratio was 1.07 (95% confidence interval 1.03–1.12) at 39 weeks of gestational and 6.18 (95% confidence interval 5.17–7.39) at gestational ages < 28 weeks. For measurement 2) “Failing to complete compulsory school” the cohort consisted of 374,798 children after exclusion of those who died, had emigrated and/or had missing neonatal data. The risk of failing to complete compulsory school increased across the entire range of gestational ages: The adjusted odds ratio was 1.07 (95% confidence interval 1.04–1.10) at 39 weeks of gestation and 2.99 (95% confidence interval 2.41–3.71) at gestational ages < 28 weeks. In both sets of analyses GA = 40 weeks was used as reference.

**Conclusions:**

We confirm a clear association between the degree of prematurity and significant school difficulties across the entire range of gestational ages from ≤ 24 to 40 weeks.

## Introduction

Preterm born children have increased risk of neurodevelopmental impairments such as cerebral palsy and severe learning disabilities appearing in the first years of life. At school age, school difficulties and academic underachievement have been extensively reported among very preterm born children (gestational age (GA) < 32 weeks) [1–3]. Further, a growing number of studies find that even children born moderate and late preterm also appear having significant school difficulties with lower cognitive abilities compared with their term born peers [4–7]. Thus, there is evidence of a stepwise increase in learning difficulties with decreasing GA rather than a threshold effect concerning the association between learning difficulties and prematurity. However, the majority of previous studies have only examined schooling among different groups of preterm born children and not across the entire range of GA’s as a continuum and the few studies that have investigated the relation as a continuum have almost exclusively investigated intelligence scores (intelligence quotients, IQ) as a proxy for school difficulties and later academic disadvantages [8–11]. However, the consequences for school performance is, still less clear.

Furthermore, accumulating evidence document that developmental adversities also appear among children born at 37–38 weeks compared to children born at 39–41 weeks [12] and it seems that these adversities persist even among children born in weeks, classified as ‘term’ (GA 37–41) [13,14]. A population-based study from Scotland investigated the relation between GA and special educational needs in school-age-children and found a stepwise increase within the entire range from 41–24 weeks of gestation [15]. However, the study from Scotland examined all kind of special educational needs (including only a few hours support weekly) and later academic achievements were not investigated.

Denmark offers good opportunities for epidemiological research given population-based registries which permit large study-populations and high follow-up rates. Our present study uses these to examine the extent of significant school difficulties among the entire range of GA’s on a population-based level. We sought to investigate both temporary, as special educational support can be, and more permanent signs of school difficulties, the latter evaluated by the risk of failing to complete compulsory school. Failing to complete compulsory school is an outcome of importance since it in Denmark is strongly associated with a high risk of not achieving a secondary education later in life [16]. For both measurements, we hypothesized that for the entire range of GA from 40 to < = 24 weeks, for every week increase in GA there is a decrease in school difficulties.

## Methods

### Study design

This study is a Danish national population-based register study. All citizens in Denmark have since 1968 [17] received a unique personal identification number, the Central Person Registry (CPR) number shortly after births. By this unique number Statistic Denmark is able to link various national registers of interest and these data can be used for the purpose of research by permission. The completeness of the registers held by Statistic Denmark is very high since the reporting to these is mandatory by law. Data access from Static Denmark can be granted to Danish researchers after a pre-approval by Statistic Denmark. Any foreign researchers must have an affiliation with a Danish authorized research-environment to be granted the approval for data extraction. All variables used in this study were extracted from registers held by Statistic Denmark in January 2017. All registers were last updated in 2015 or 2016.

### Definition of school difficulties

School difficulties were investigated by two different measurements 1) Special educational support in compulsory school and 2) Failing to complete compulsory school. These two endpoints were chosen since they both represent compound outcomes influenced by IQ but also other aspects of schooling such as attention deficits, social problems and health stressors. Further, failing to complete compulsory school is of significant importance in a Danish context since it is a strong predictor for not achieving further educational qualifications.

“Special educational support”: In Denmark children with school difficulties can receive special educational support in compulsory school either as supportive measures in the general class or in a special class. Based on a pedagogical and psychological assessment the headmaster of the school, in consultation with the parents and the pupil, decides whether the pupil must receive special education [18]. It is mandatory by law that the school administration reports data on special educational support to the individual pupil to the national Danish special educational register once a year. Children receiving at least 9 hours special educational support weekly are registered. For the present analyses data from the special educational register were collected and special educational support was defined if a pupil was registered with special education and thereby received at least 9 hours support weekly.

“Failing to complete compulsory school”: In Denmark, all children have compulsory education in the period from the first of August the calendar year of the 6^th^ birthday till the ninth grade is completed or till the 31^th^ of July the calendar year of the 17^th^ birthday [19]. It is possible to delay the beginning of the education one year with permission. Thus, most children begin school at 5 to 6 years of age where the public-sector school starts at zero grade and ends after 10 years of compulsory school with the examination after ninth grade typically at the age of 15 to 16 years. Almost all children receive compulsory education in either a public-sector school (approximately 80%) or a private school (approximately 16%) [20]. The examinations after ninth grade are identical across the country covering major domains where the Ministry of Education has decided that exams in Danish and mathematics are mandatory exams to attend for all pupils leaving compulsory school. Only a few private schools, representing approximately 1% [21,22], along with some specific schools for children with severe learning difficulties, have permission not to offer examinations after the ninth grade. It is mandatory that the administrations of the schools report the results from the examination after 9^th^ grade to the databases of Compulsory School Grades and these data were collected for the present analysis.

In the present study pupils were registered as having failed to complete compulsory school if the pupil had not attended the mandatory exams in Danish and mathematics at the final examination after ninth grade before the 18^th^ birthday.

### The study populations

The study population differed in the two sets of analyses according to the two measurements “Special educational support” and “Failing to complete compulsory school”.

“Special educational support”: This study population was generated from the special educational register which includes all individuals in compulsory school from zero to ninth grade in the school-year 2015/2016.

“Failing to complete compulsory school”: This study population was generated from the Medical Birth register and included all live born infants born in Denmark in the 6-year period from 1992 to 1997. Survival rates and emigration status were obtained using the Cause of Death register and the Migration register.

Covariates with potential confounding influence on the association between GA and school difficulties were achieved in various registers held by statistic Denmark.

Data on GA at delivery, birth weight, gender, mode of delivery, and multiple births were obtained from the Medical Births register. To make the study as representative of the whole population as possible, all individuals were included if these data were available. For all infants in the cohorts the birth weight was adjusted for gestational age at delivery and thereby recalculated to a birth weight standard deviation score (SDS), a z-score, according to gender and GA by methods described by Marsal [23]. The birth weight SDS was divided in four groups: below -3 SDS, from -3 to -2 SDS, from -2 to -1 SDS and above -1 SDS.

Information on highest achieved level of education on both parents recorded at the time of childbirth was obtained from the Educational register. The educational level was categorized in three groups (high, middle and low) based on the International Standard Classification of Education (ISCED) and the parent with the highest educational level was used to define the educational level of the parents. The three groups of educational levels were defined according to ISCED as: low ISCED 0–2, middle ISCED 3–5 and high ISCED 6–8.

For both measurements of school difficulties, we excluded children with missing information on GA and/or birth weight and to minimize the risk of miscoded cases, also children with birth weight SDS value outliers (+/- 6 SDS). Furthermore, we excluded children with a GA below 21 weeks or above 44 weeks.

### Statistical analyses

The relationship between GA and school difficulties was investigated in two sets of regression analyses with the two outcomes “Special educational support” and “Failing to complete compulsory school” as defined.

In both sets of analyses following covariates were included: gender, plurality, birth weight SDS groups, mode of delivery and the parents’ educational level. GA, measured in completed weeks, was gathered in 8 groups to facilitate the presentation of the analyses. Both sets of analyses were performed in two steps–First; a univariate logistic regression analyses investigated the association between the chosen explanatory variables and the measurement of school difficulties, Second; a multiple logistic regression analyses investigated the association between GA and school difficulties adjusted for the effect of the chosen covariates. In both sets of analyses GA = 40 weeks was used as reference. A p-value ≤ 0.05 was considered statistically significant.

Further, the extent of pupils who failed to complete compulsory school according to GA was described by estimating the excess risks. The GA was divided in groups and the excess risk was calculated by calculating the attributable risk and the population attributable risk to investigate the extent at the individual and the community level respectively.

The SAS 9.4 statistical package was used.

### Ethics

The study is through a wide approval held by Statistic Denmark approved by The Danish Data Protection Agency. Further approval from the Ethics committee or consent from each single individual is not necessary in Denmark with this type of study-design. Data were only available in an anonymous form, and all processing was run on the computers of Statistic Denmark.

## Results

**Fig 1:**
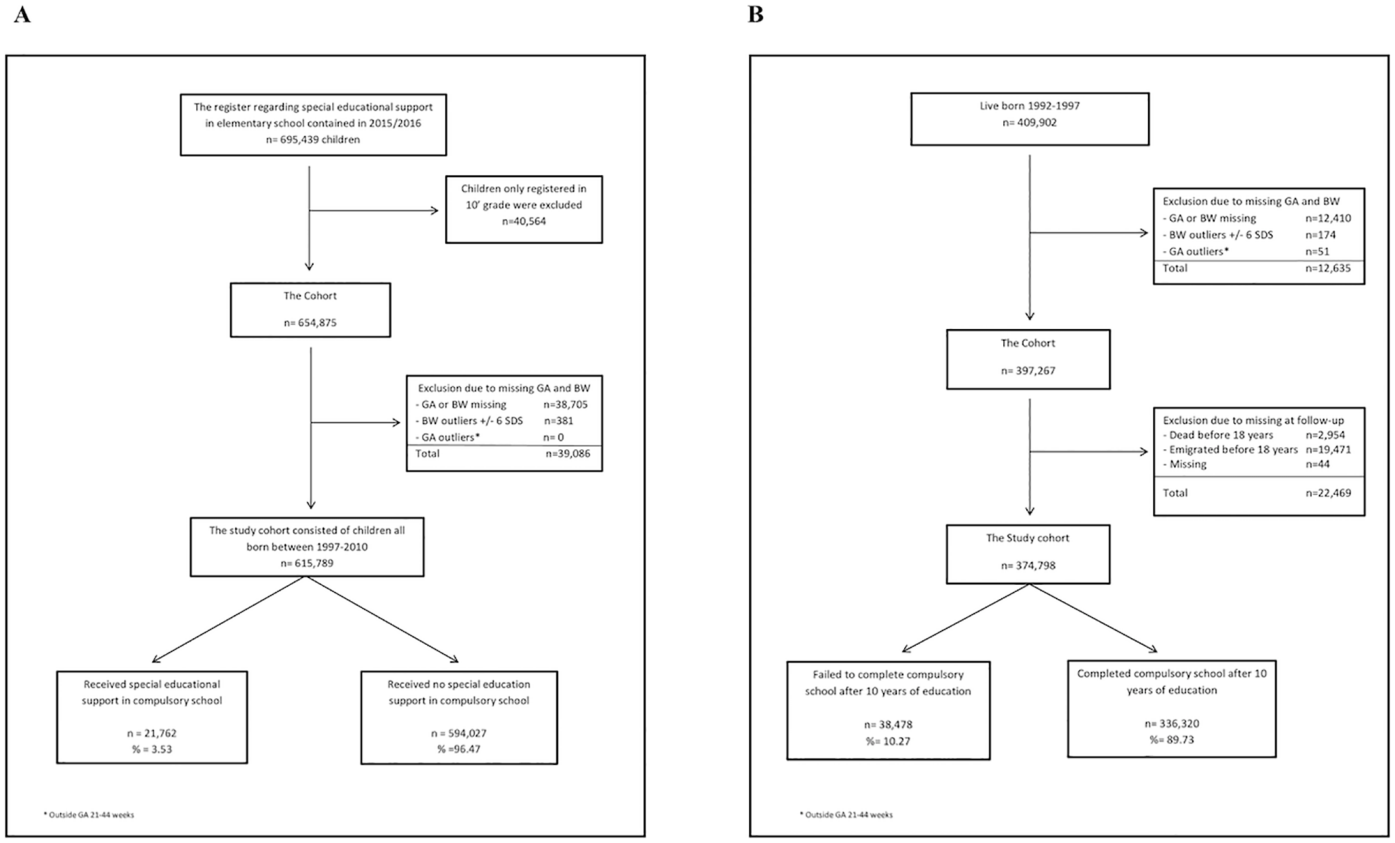
The two study populations of children who (A) were registered in a Danish compulsory school in the school year 2015/2016 and (B) were born in 1992–1997. GA indicates gestational age, BW, birth weight and SDS, standard deviation score for birth weight by gestation.

For the analyses concerning special educational support there were 695,439 children registered as pupils in the compulsory schools in Denmark in the school year 2015/2016. After exclusion of children with missing neonatal-data (n = 39,086, 5.6%) and children who were only registered in 10^th^ grade (n = 40,564, 5.8%), 88.6% (n = 615,789) of all children in compulsory school in Denmark in the school year 2015/2016 were included in the analyses (Fig 1A). In the study population 5.5% (n = 33,786) were born preterm (GA<37 weeks) and 3.5% (n = 21,762) received comprehensive special educational support more than 9 hours weekly. For the analyses concerning “failing to complete compulsory school” 409,902 live born children were registered between 1992 and 1997 in the Medical Birth register. After exclusion of the deceased (n = 2,954, 0.7%), the emigrated (n = 19,471, 4.8%) and those with missing neonatal-data (n = 12,679, 3.1%), 91.4% of children (n = 374,798) born in Denmark in the period 1992–1997 were included in the analyses (Fig 1B). The GA distribution of the emigrated children was almost identical with the GA distribution in the rest of the cohort. In the study population 5.5% (n = 20,596) were born preterm (GA<37 weeks) and a total of 10.3% (n = 38,478) failed to complete compulsory school.

**Fig 2:**
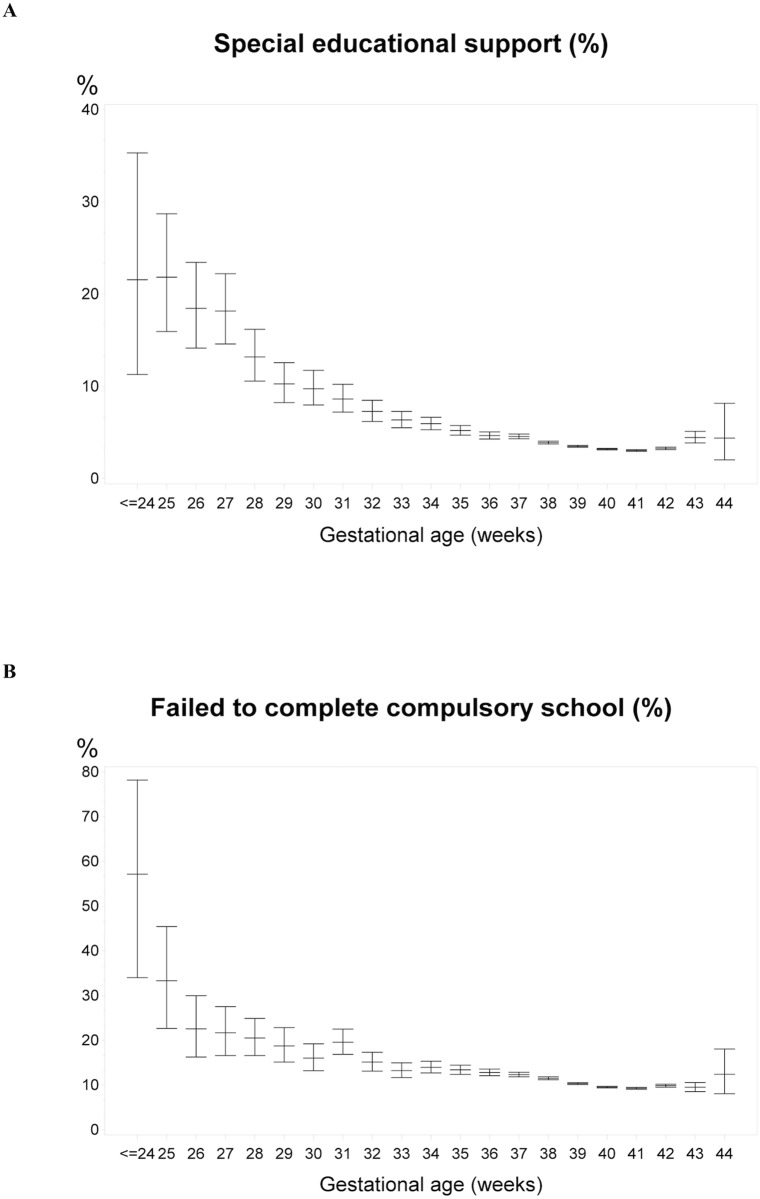
The percentage distribution of children who (A) received special educational support in compulsory school and (B) failed to complete compulsory school, by gestational age at birth.

GA showed a clear dose-effect association as regard to special educational support as well as failing to complete compulsory school (Fig 2). For both measurements, the risk of school difficulties steadily increased with decreasing GA from 41 to < = 24 weeks, thus the percentage of children with special educational support increased from 3.0% (95% confidence interval (CI): 3.0–3.1) at GA = 41 weeks to 21.6% (95% CI: 11.3–35.3) at GA< = 24 weeks. Similar the proportion of those who did not complete compulsory school increased from 9.3% (95% CI: 9.1–9.5) at 41 weeks to 57.1% (95% CI: 34.0–78.2) at GA< = 24 weeks. For both measurements, a slightly increased risk was found among children born post-term, GA > = 42 weeks (Fig 2).

In both the univariate and the multivariate logistic regression analyses the parents’ educational level and the birth weight SDS-groups were strongly associated with both measurements of school difficulties in a dose-effect like relationship (Tables 1 and 2). In the multiple logistic regression analyses the risk of school difficulties increased significantly across the range of gestation from 41 to < 28 weeks. For special educational support, the adjusted OR was 1.07 (95% CI 1.03–1.12) at 39 weeks increasing gradually to OR 6.18 (95% CI 5.17–7.39) in the group of children with GA < 28 weeks (Table 1). For failing to complete compulsory school, the adjusted OR was 1.07 (95% CI 1.04–1.10) at 39 weeks gradually increasing to OR 2.99 (95% CI 2.41–3.71) in the group of children with GA < 28 weeks (Table 2). For the post-term children (GA> = 42 weeks) the OR were not statistically significant compared with children born at GA = 40 weeks (Tables 1 and 2).

**Table 1:** Uni- and multivariate logistic regression analyses with related odds ratios for provision of special educational support for all children registered in the Danish compulsory school in the school-year 2015/2016.

Label	Logistic regression analysis Received special education support (n = 21,762; 3.53%)	
	Univariate analysis Odds Ratio (95% CI) n = 615,789^1^	Multivariate analysis Odds Ratio (95% CI) n = 612,389
**Gestational age (weeks)** *< 28 (n = 930; 0.15%) vs 40*	7.18** (6.09–8.48)	6.18** (5.17–7.39)
*28–31 (n = 3,798; 0.62) vs 40*	3.36** (3.01–3.75)	2.64** (2.35–2.98)
*32–34 (n = 9,943; 1.61%) vs 40*	2.06** (1.89–2.24)	1.73** (1.58–1.90)
*35–36 (n = 19,115; 3.10%) vs 40*	1.55** (1.45–1.67)	1.40** (1.30–1.51)
*37–38 (n = 89,528; 14.54%) vs 40*	1.30** (1.24–1.35)	1.23** (1.18–1.29)
*39 (n = 115,369; 18.74%) vs 40*	1.10** (1.06–1.15)	1.07* (1.03–1.12)
*40 (n = 162,169; 26.34%)*	1	1
*41 (n = 138,712; 22.53%) vs 40*	0.95* (0.91–0.99)	0.97 (0.93–1.01)
*> = 42 (n = 76,225; 12.38%) vs 40*	1.05 (1.00–1.10)	1.02 (0.97–1.07)
**Gender** *Male (n = 316,215; 51.35%) vs Female*	2.75** (2.67–2.84)	2.82** (2.73–2.90)
**Section** *Yes (n = 126,968; 20.62%) vs No*	1.22** (1.18–1.26)	1.09** (1.05–1.13)
**Multiple births** *Yes (26,199; 4.25%) vs No*	1.04 (0.97–1.11)	0.67** (0.62–0.72)
**Birth weight SDS**^2^ *-1SDS to -2 SDS (n = 89,586; 14.55%) vs above -1SDS*	1.45** (1.40–1.50)	1.35** (1.30–1.40)
*-2SDS to -3SDS (n = 16,991; 2.76%) vs above -1SDS*	2.33** (2.20–2.48)	1.96** (1.84–2.08)
*Below -3 SDS (n = 2,619; 0.43%) vs above -1SDS*	4.20** (3.73–4.73)	2.74** (2.41–3.12)
**Parents education** *Low (n = 55,273; 9.03%) vs High*	5.68** (5.46–5.92)	5.44** (5.23–5.67)
*Middel (n = 296,531; 48.42%) vs High*	2.11** (2.04–2.18)	2.06** (1.99–2.14)

** p<0.0001

* p<0.05

^1^ All analyses included 615,789 children except the analyses regarding parent’s educational level where only 612,389 children were included.

^2^ SDS indicates standard deviation score for birth weight by gestation.

**Table 2:** Uni- and multivariate logistic regression analyses with related odds ratios for failing to complete compulsory school after 10 years of education for children born in 1992–1997.

Label	Logistic regression analysis Failing to complete compulsory school (n = 38,478; 10.27%)	
	Univariate analysis Odds Ratio (95% CI) n = 374,798^1^	Multivariate analysis Odds Ratio (95% CI) n = 373,457
**Gestational age (weeks)** *< 28 (n = 483; 0.13%) vs 40*	3.21** (2.61–3.94)	2.99** (2.41–3.71)
*28–31 (n = 2,214; 0.59) vs 40*	2.17** (1.94–2.42)	1.76** (1.56–1.97)
*32–34 (n = 5,678; 1.51%) vs 40*	1.54** (1.43–1.67)	1.31** (1.20–1.42)
*35–36 (n = 12,221; 3.26%) vs 40*	1.42** (1.34–1.50)	1.26** (1.18–1.33)
*37–38 (n = 56,180; 14.99%) vs 40*	1.26** (1.22–1.31)	1.18** (1.14–1.22)
*39 (n = 77,849; 20.77%) vs 40*	1.09** (1.06–1.13)	1.07** (1.04–1.10)
*40 (n = 114,972; 30.68%)*	1	1
*41 (n = 70,281; 18.75%) vs 40*	0.97* (0.94–1.00)	0.97* (0.94–1.00)
*> = 42 (n = 34,920; 9.32%) vs 40*	1.03 (0.99–1.08)	0.97 (0.93–1.02)
**Gender** *Male (n = 192,665; 51.41%) vs Female*	1.60** (1.57–1.63)	1.63** (1.60–1.67)
**Section** *Yes (n = 76,123; 20.31%) vs No*	1.18** (1.15–1.21)	1.10** (1.07–1.13)
**Multiple births** *Yes (n = 12,440; 3.32%) vs No*	0.96 (0.91–1.02)	0.70** (0.65–0.75)
**Birth weight SDS**^2^ *-1SDS to -2SDS (n = 52,490; 14%) vs above -1SDS*	1.40** (1.36–1.44)	1.30** (1.26–1.34)
*-2SDS to -3SDS (n = 11,316; 3.02%) vs above -1SDS*	1.98** (1.89–2.08)	1.71** (1.62–1.80)
*Below -3SDS (n = 1,878; 0.5%) vs above -1SDS*	2.68** (2.40–2.99)	2.14** (1.90–2.41)
**Parents education** *Low (n = 52,021; 13.93%) vs High*	5.86** (5.67–6.06)	5.66** (5.48–5.86)
*Middel (n = 212,592; 56.93%) vs High*	1.94** (1.88–2.00)	1.91** (1.85–1.97)

** p<0.0001

* p<0.05

^1^ All analyses included 374,798 children except the analyses regarding parent’s educational level where only 373,457 children were included.

^2^ SDS indicates standard deviation score for birth weight by gestation.

The excess risks for extremely and very preterm born infants (the attributable risks) of failing to complete compulsory school were respectively 16 and 9 per 100 children compared with 2 per 100 children for early term born infants (Table 3). At the population level, however, the excess risk of failing to complete compulsory school were much higher for the majority of children (728 per 100,000) born early term compared to the 66 and 171 per 100,000 when looking at the extremely and very preterm born infants (Table 3).

**Table 3:** The attributable risk and population attributable risk for failing to complete compulsory school after 10 years of education. Week 40 is used as reference.

**Label GA** **(weeks)**	**Attributable risk pr. 100 children**	**Population attributable risk pr. 100,000 children**
Extremely preterm (< 28)	15.7	65.8
Very preterm (28–31)	9.1	171.3
Moderate preterm (32–34)	4.5	209.9
Late preterm (35–36)	3.5	334.2
Early term (37–38)	2.2	728.1
Term (39)	0.8	314.7
Term* (41)	-0.3	-102.1
Post-term (> = 42)	0.3	67.7

* Since week 40 is used as reference but children born in week 41 have a lower risk of failing to complete compulsory school the excess risk is negative for this week.

## Discussion

In our large population-based study we found that ex-preterm children have an increased risk of school difficulties measured as special educational support and failing to complete compulsory school. Further the association between significant school difficulties and GA increased with decreasing GA from 41 to 24 weeks of gestation including the GA’s classified as “term” (GA 37–41). We investigated the two outcome-measures of school difficulties by two different study-populations and were therefore not able to investigate the specific interaction between the need for special educational and lack of completion of compulsory school. Despite this, the results regarding the two outcomes were very similar with both outcomes showing signs of a similar dose-effect like relationship. Our study therefore indicates that an increased risk of special educational support, not only is a marker of temporary school difficulties since a related association was found for failing to complete compulsory school. These are important outcomes of practical importance. Special education at a level of 9 hours or more weekly is costly, and failing to complete compulsory school has direct implications for further educational possibilities since it predicts a lower chance of receiving further secondary education and thereby predicts a lower final educational level [16]. This is in accordance with a previous study from Denmark comparing 1,422 individuals born at GA<33 weeks with nearly 200,000 term born individuals at age 27–29 years [24]. The rate of individuals with only compulsory school as the final education level was 8% higher in the preterm group and the rate of academic education was 5.7% lower.

There are only few studies investigating the association between GA as a continuum among the entire range of GA [15,25] and school difficulties and even fewer investigating final school performance. A large register-based cohort study from Sweden [25], evaluating 1,643,958 children on school performance measured in grades in compulsory school according to GA found a strong negative association between GA and school performance. However, compared to matched siblings the association vanished completely for infants born with a GA = 31 weeks or more. This contrasts with our study in which we used the parental educational level to adjust for genetic and social-environmental factors, however, the discrepancy could be explained by a selection bias in follow-up in the Swedish study since only the pupils that obtained school grades were included in the analyses. Our study corroborates the results from the majority of previous follow-up studies [5,15,26–28] and case-control studies from several different countries concerning very, early and late preterm born children [1,3,29]. Further, in recent years studies comparing early and late full-term born infants [7,30] also found a significant association concerning increased cognitive impairment with decreasing GA like our study. Thus, the dose-effect association between school difficulties and GA seems multinational and suggests an underlying neurodevelopmental cause rather than only childhood environmental factors. The brain is one of the last fetal organ systems to mature and since the last half of gestation is considered a critical period for brain development this could be the explanation for our findings [31]. Alterations in brain development associated with both very and late preterm birth have been found in MR studies and some of these alterations are associated with long-term development of the children [32,33]. A number of intra- and extra-uterine factors related to a shortened pregnancy and prematurity are likely to have causative roles. Of these, we only investigated the effect of birth weight and found a strong association between the birth weight SDS and school difficulties. Birth weight SDS is a marker of intrauterine pathological processes associated with brain development, rather than a cause in itself.

The associations for both special educational support and failing to complete compulsory school were strongest at the lowest GA. The majority of even the smallest infants actually completed compulsory school, but at the individual level the excess risk for these infants is high and has implications for follow-up, support and intervention for the children and their families. On the contrary, the excess risks for late preterm and early term infants were small compared to the influence of the social, environmental and genetic factors measured as parental educational level in our study. At the community level, however, late preterm and early term born infants accounts for a much higher proportion of the population with school difficulties. Our findings may even be used as support for the recent recommendations concerning timing of elective cesarean section on maternal request to be planned not before 39 weeks of gestation [34].

Our study has several strengths. It is a national population-based study and thus unselected. It is the largest study of its kind to investigate the extent of significant school-difficulties among the entire range of GA’s. All data were obtained from routine data from the Danish national registers which has a high level of completeness since several data used in this study is mandatory by law to report to Statistic Denmark. By linking national registers and using data as both inclusion- and outcome-measures we ensured that bias related to loss of follow-up was minimized. Thus, in our study-population regarding failing to complete compulsory school, 95% of the birth cohort was traced to follow-up at age 18 years. By the inclusion of this considerable number of children, the study is suitable to detect minor differences between groups as seen for the late preterm and early term born infants. On the contrary, this study-design only has a limited amount of information available and therefore it was not possible to describe why pupils received special educational support or specific reasons for not completing compulsory school. In our analyses, we adjusted for parental educational level, which served as an indicator for genetic and social environmental factors but residual confounding is very likely, although unlikely to change the conclusions significantly. Thus, the most important weakness of our study is the limited amount of information and the unmeasured confounders.

## Conclusion

We confirm a clear association between the degree of prematurity and significant school difficulties across the entire range of GA’s. At the community level, the late preterm and early term born infants who failed to complete compulsory school after 10 years of education far outnumber the very preterm born infants.

## Supporting information

S1 Fig Deaths in the cohort (1992–1997) by gestational age. (TIF)

S2 Fig Mean birth weights for the two study populations. (TIF)

## Abbreviations

CI: confidence interval
CPR: central person registry
GA: gestational age
IQ: intelligence quotient
ISCED: international standard classification of education
OR: odds ratio
SDS: standard deviation score
