# Supplementary figures and images for: Low gestational age at birth and difficulties in school—A matter of ‘dose’
Source: PLoS One. 2018 Jun 20;13(6):e0198482. doi: 10.1371/journal.pone.0198482 (PMC6010215; doi:10.1371/journal.pone.0198482)

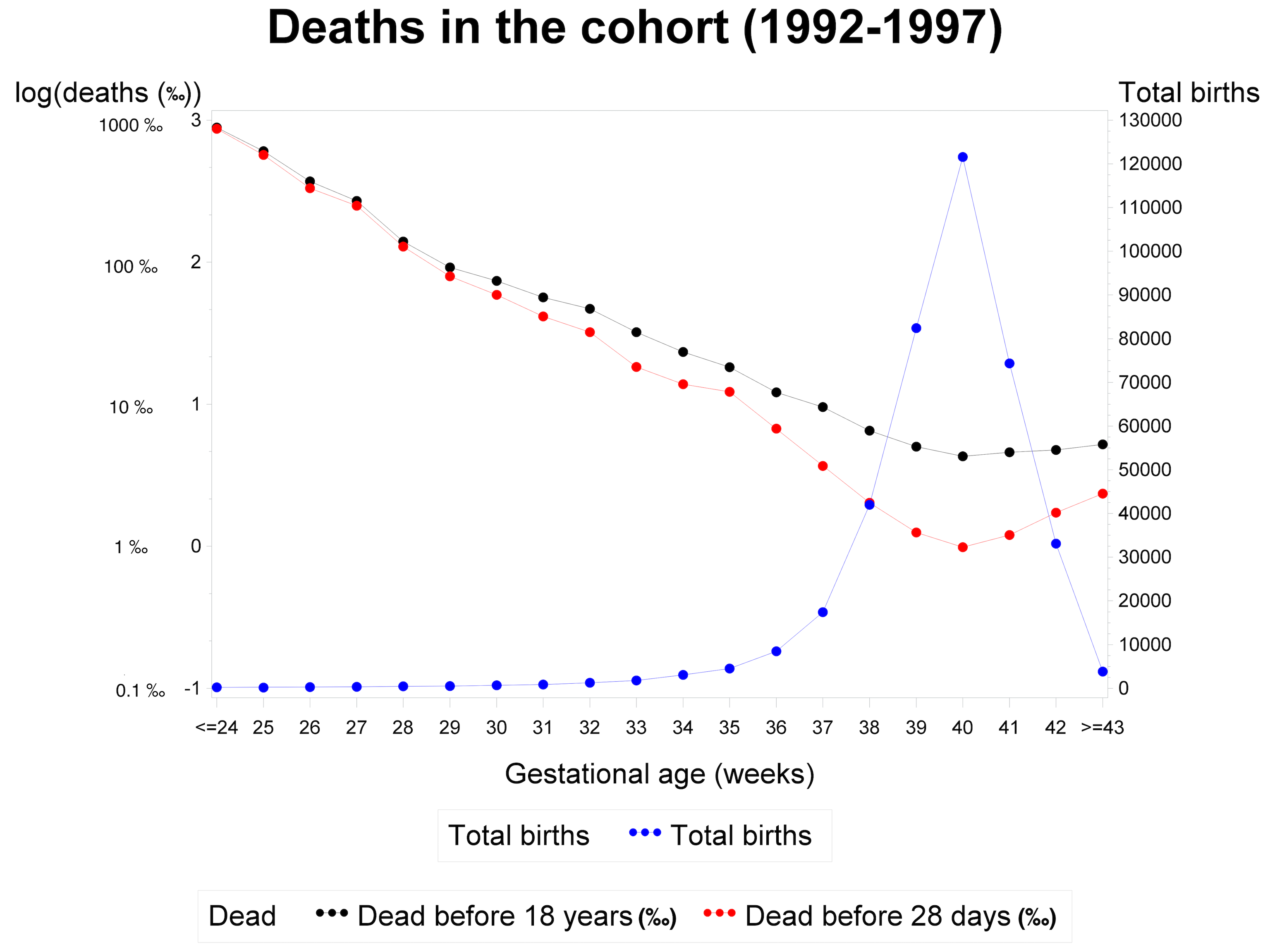

Supplement: S1 Fig — (TIF) [file pone.0198482.s001.tif]

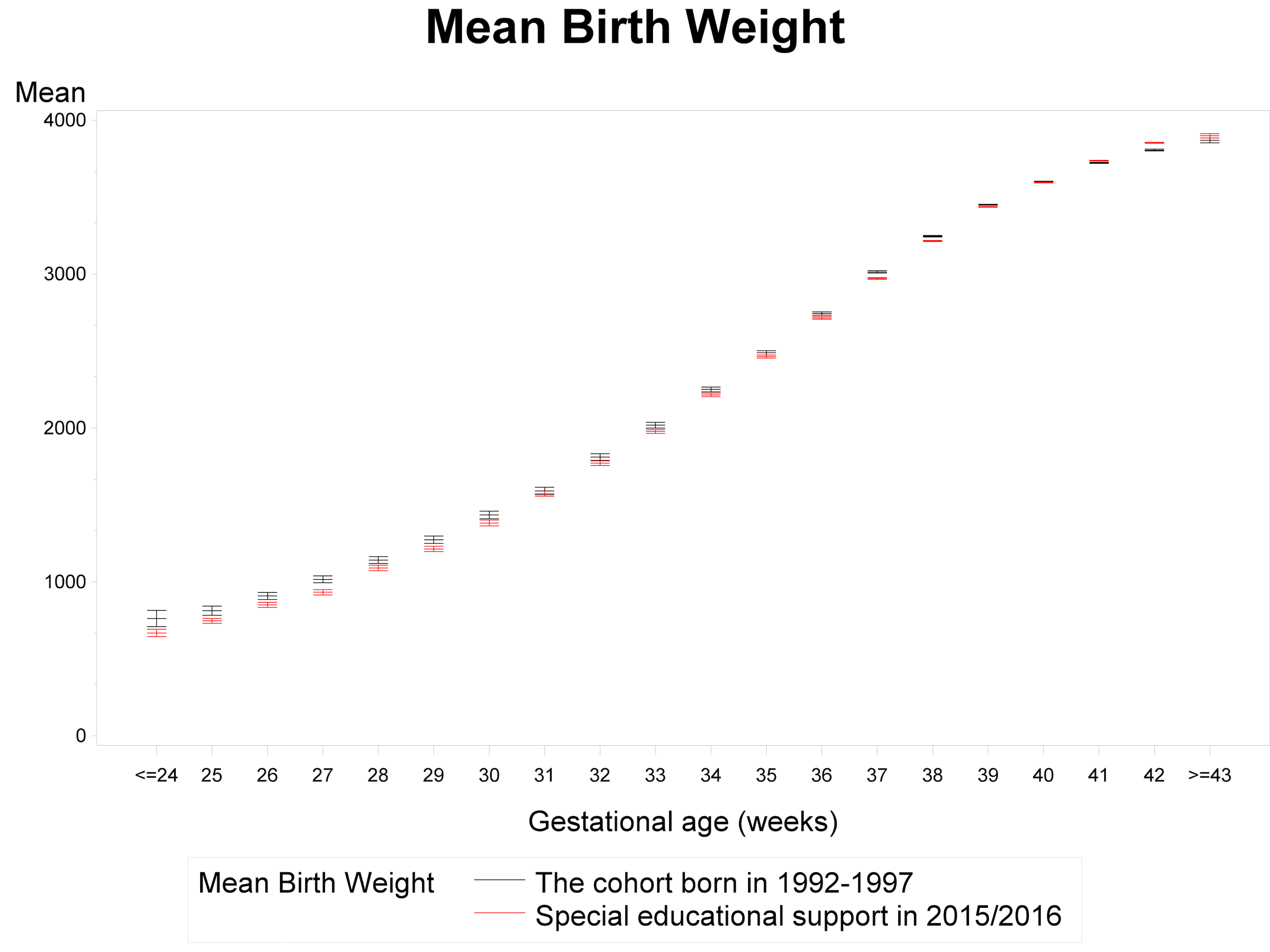

Supplement: S2 Fig — (TIF) [file pone.0198482.s002.tif]
